# Family Caregivers of Veterans Experience High Burden, Mental Health Distress, and Financial Strain

**DOI:** 10.1093/geroni/igaa057.2499

**Published:** 2020-12-16

**Authors:** Megan Shepherd-Banigan, Sophia Sherman, Jennifer Lindquist, Katherine Miller, Matthew Tucker, Valerie Smith, Courtney Van Houtven

**Affiliations:** 1 Duke University School of Medicine, Durham, North Carolina, United States; 2 US Department of Veterans Affairs HSR&D, Durham, North Carolina, United States; 3 U.S. Department of Veterans Affairs HSR&D, Durham, North Carolina, United States; 4 US Department of Veterans Affairs, Durham, North Carolina, United States

## Abstract

We describe the caregiving experiences and needs of family caregivers of older Veterans enrolled in the U.S. Department of Veterans Affairs (VA). We conducted telephone surveys with 1,509 caregivers to assess caregiver health and well-being. Caregivers were primarily female, <50 years old, white, and the Veterans’ spouse. Veterans had substantial functional limitations and required care for multiple conditions, commonly, mental illness, dementia, and heart disease. On average, caregivers provided care for 9.6 hours per day and 6.7 days per week. Burden and depressive symptoms were above clinical thresholds with average scores of 21.8 (Zarit burden) and 11.5 (CES-D 10). Levels of perceived loneliness and financial strain were high. As this population needs emotional support, respite care services, social engagement, and training to care for aging Veterans, the expansion of enhanced caregiver services and supports to this population (expected in 2020) through the VA Mission Act of 2018 will be beneficial.

